# Analysis of Alzheimer's disease mortality in Brazil between 2020 and 2023 by age group and gender

**DOI:** 10.1002/alz70860_107636

**Published:** 2025-12-23

**Authors:** Vitor Ritt Xavier, Ana Carolina Sgarioni Viapiana, Morghana Machado da Rosa, Rodrigo de Cezaro, Cavaler Juvêncio, Vitório Serafim, Gabrielle Guindani Maia, Leonardo Bedatti Koehler, Ana Júlia Rodrigues Ribeiro, Jessica Vargas Lopes, Laura Carolina Nardi Motta

**Affiliations:** ^1^ Fundação Universidade Federal de Ciências da Saúde de Porto Alegre, Porto Alegre, Rio Grande do Sul, Brazil; ^2^ Universidade Federal do Rio Grande do Sul, Porto Alegre, Rio Grande do Sul, Brazil; ^3^ Universidade Luterana do Brasil, Canoas, Rio Grande do Sul, Brazil

## Abstract

**Background:**

In Brazil, 1.2 million people are living with Alzheimer's Disease (AD), according to the Brazilian Alzheimer's Association and the Brazilian Hospital Services Company, and approximately 100.000 new cases are diagnosed per year. In this context, analyzing the distribution of AD‐related deaths is essential for shaping public health policies. Therefore, this study seeks to compare Brazilian public health data on AD, examining the age and gender distribution of AD‐related deaths from 2020 to 2023.

**Method:**

This is a cross‐sectional study of epidemiological data on AD mortality in the Brazilian population between 2020 and 2023. The data, obtained from the Information Technology Department of the Unified Health System, were stratified by gender and age groups (40–49, 50–59, 60–69, 70–79, and 80 or older).

**Result:**

From 2020 to 2023, 108.768 deaths were attributed to AD and to AD Related Dementia (ADRD). Approximately 77% of them occurred in individuals aged 80. Throughout that period, there was a fivefold increase in mortality among females when compared those aged 80 and older to the 70‐79 age group. There was only a 2.87‐fold increase in the same age group among males. In the 60 to 69 age group, the mortality ratio of women to men was only 1.03, which reveals a minimal difference between them. However, considering the age groups over 70, the ratio rises to 1.88, reinforcing that, in older age, women are at higher risk of dying of AD and ADRD.

**Conclusion:**

Brazil, following the global trend, registered increasing mortality rates due to AD and ADRD. The results, aligned with previous findings, reveal that females aged 70 or older face the highest risk of dying of AD. The available data are insufficient to determine whether the increased mortality of women aged 80 or older is attributable to a greater probability of AD development or to a tendency to experience more severe forms of the disease when it is diagnosed. Thus, enhancing the public statistical data system is essential to support effective public health policies, especially because, as observed here, AD represents a significant burden of disease, death and disability.

